# Novel Reassortant Avian Influenza A(H5N6) Viruses in Humans, Guangdong, China, 2015

**DOI:** 10.3201/eid2208.160146

**Published:** 2016-08

**Authors:** Yong-Yi Shen, Chang-Wen Ke, Qian Li, Run-Yu Yuan, Dan Xiang, Wei-Xin Jia, Yun-Di Yu, Lu Liu, Can Huang, Wen-Bao Qi, Reina Sikkema, Jie Wu, Marion Koopmans, Ming Liao

**Affiliations:** Key Laboratory of Zoonosis Prevention and Control of Guangdong Province, Guangzhou (Y.-Y. Shen);; College of Veterinary Medicine, South China Agricultural University, Guangzhou, China (Y.-Y. Shen, Q. Li, W.-X. Jia, Y.-D. Yu, W.-B. Qi, M. Liao);; Guangdong Provincial Center for Disease Control and Prevention, Guangzhou (C.-W. Ke, R.-Y. Yuan, J. Wu);; Shantou University Medical College, Shantou, China (D. Xiang, L. Liu, C. Huang);; Key Laboratory of Veterinary Vaccine Innovation of the Ministry of Agriculture, Guangzhou (W.-B. Qi);; National Institute of Public Health and the Environment, Bilthoven, the Netherlands (R. Sikkema, M. Koopmans);; National and Regional Joint Engineering Laboratory for Medicament of Zoonosis Prevention and Control, Guangzhou (M. Liao)

**Keywords:** H5N6, avian influenza, A(H5N6), novel, reassortant, viruses, zoonoses, Guangdong, China, influenza

**To the Editor:** Avian influenza A(H5N6) influenza viruses have circulated among poultry in southern (Jiangxi, Guangdong) and western (Sichuan) provinces of China since 2013 (*1*,*2*). In 2014, outbreaks of H5N6 virus infection occurred among poultry in China, Laos, and Vietnam (*1*). In April 2014, the first case of highly pathogenic H5N6 infection among humans was detected in Sichuan Province (*3*); the second case was detected on December 3, 2014, in Guangdong Province (*4*). In December 2015, 4 humans in Guangdong Province were infected with H5N6 influenza (*5*,*6*).

To study the genetic basis of continuing human infections with this avian influenza subtype, we sequenced the complete genomes of 2 of the 4 human H5N6 isolates obtained in December 2015 in Guangdong Province. We compared these sequences with those of 1 H6N6 and 8 H5N6 influenza viruses isolated from birds in live poultry markets in this region during 2013–2015 (Technical Appendix) and other published genomes of H5, H6N6, and H9N2 avian influenza viruses (Technical Appendix). Phylogenetic analyses of the hemagglutinin (HA) genes showed that all human H5N6 isolates belonged to clade 2.3.4.4 (Technical Appendix Figure 1, panel A). HA and neuraminidase (NA) genes of some H5N6 viruses isolated in Guangdong Province during 2013–2014 were in the Sichuan-like lineage, but all of those from 2015 were in the Jiangxi-like lineage (Technical Appendix Figure 1, panels A, B).

Despite the similarities of the HA and NA genes, the 6 internal genes from the 2 human isolates from 2015, A/Guangdong/ZQ874/2015 (H5N6) and A/Guangdong/SZ872/2015 (H5N6) were different from 2 human H5N6 isolates from 2014, A/Sichuan/26221/2014 (H5N6) and A/Guangzhou/39715/2014 (H5N6). The polymerase basic (PB) 2 gene from isolate A/Guangdong/ZQ874/2015 (H5N6) appears to have derived from an H6N6 virus isolated from a duck; all other genes in this isolate were derived from H5N6 viruses that have been circulating among poultry since 2013 (Technical Appendix Figure 1, panel C; Technical Appendix Figure 2). This isolate showed high nucleotide identity to 6 of the 8 genes (HA, 96.5%; NA, 98.2%; nucleoprotein (NP), 98.5%; polymerase acidic (PA), 98.3%; PB1, 98.1%; PB2, 98.4%) of the isolate A/chicken/Guandong/FG594/2015 (H5N6); the identities of the matrix (M) and nonstructural protein (NSP) genes were 76.2% and 79.8% similar, respectively. This finding suggests that undetected reassortants of H5N6 may exist. The other human isolate, A/Guangdong/SZ872/2015 (H5N6), showed high nucleotide identity with A/Yunnan/0127/2015 (H5N6), an isolate collected from a person in Yunnan Province (GenBank accession nos. KT963053–60; Technical Appendix Table), for all 8 genes (HA, 97.2%; M, 97.7%; NA, 96.8%; NP, 98.3%; NSP, 93.2%; PA, 95.9%; PB1, 96.9%; PB2, 94.0%). The 6 internal genes of A/Guangdong/SZ872/2015 (H5N6) appear to have come from the enzootic H9N2 (ZJ-HJ/07) virus lineage (Technical Appendix Figure 1, panel C). These findings show that the circulating H5N6 virus in southern China has reassorted with enzootic H6N6 and H9N2 viruses, resulting in new H5N6 viruses that are capable of infecting humans.

We compared the 2 newly sequenced genomes with 3 available genomes of human influenza virus strains in public databases to determine if they had attained key molecular features associated with increased virulence in mammals, mammalian transmissibility, and antiviral resistance (Table). The HA gene cleavage site encoded by all 5 isolates from humans showed a multiple basic amino acid motif (REKRRKR↓G), which indicates high pathogenicity in poultry. The viruses isolated from humans in 2014 had no mutations associated with reduced sensitivity to adamantine antiviral drugs, but 2 of the 3 viruses isolated in 2015 have the 31N amino acid in M2, suggesting that those 2 viruses have acquired resistance. Thus, this virus lineage could be a great threat to public health.

**Table:** Molecular analyses of 5 influenza A(H5N6) virus isolates from humans in China, 2014 and 2015*

Phenotypic consequences	Mutations	A/Sichuan/ 26221/2014	A/Guangzhou/39715/2014	A/Yunan/ 0127/2015	A/Guangdong/SZ872/2015	A/Guangdong/ZQ874/2015
HA gene						
Altered receptor specificity	S128P	T	P	P	P	P
Increased α2,6-SA recognition	S137A	A	A	A	A	A
Removal of the 158N glycosylation	T160A	A	T	A	A	A
	Q226L	Q	Q	Q	Q	Q
Cleavage site sequence	Not applicable	REKRRKR↓G	REKRRKR↓G	REKRRKR↓G	REKRRKR↓G	REKRRKR↓G
NA gene						
59–69 del	TIINNHPQNNF	No	Yes	Yes	Yes	Yes
Oseltamivir resistance	H274Y	H	H	H	H	H
	N294S	N	N	N	N	N
PB2						
Increased pathogenicity in mice	L89V	V	V	V	V	V
	E627K	E	K	K	E	E
Increased virulence and replication in mice	G309D, T339K R477G, I495V	DMGV	DTGV	DKGV	DKGV	DKGV
Enhanced transmission	D701N	N	D	D	D	N
NS1						
Increased virulence in mice and pigs	D92E	E	E	D	D	E
PDZ-motif	Not applicable	ESEV	ESEV	KPEV	KPEV	ESEV
Increased virulence in mice	P42S	S	S	S	S	S
M2						
Antiviral resistance (amantadine)	S31N	S	S	N	N	S

*HA, hemagglutinin; M2, matrix protein 2; NA, neuraminidase; NS1, nonstructural protein 1; PB2, polymerase basic 2 amino acid.

Although H9N2 is not highly pathogenic in poultry, it provides internal genes for the recent emergence of many novel avian influenza viruses that infect humans, such as the H5N6 virus in this study, as well as the H7N9 (*7*,*8*) and H10N8 (*9*) viruses. Infection with H6 subtype viruses results in no clinically significant signs of disease in poultry (*10*), but co-circulation of these viruses with other subtypes among poultry results in transfer of internal genes. This reassortment has resulted in a major increase in genetic diversity among the H5N6 viruses that cause human infections; therefore, low-pathogenicity viruses in poultry should also be controlled in poultry.

In summary, we isolated 2 novel reassortant H5N6 viruses from 2 patients in Guangdong Province, China. The internal genes of these strains are different from those found in the first wave of H5N6 infections in 2014. The PB2 of 2 human isolate A/Guangdong/ZQ874/2015 (H5N6) appears to have been derived from a duck H6N6 virus, and all other genes of this virus originated in circulating H5N6 viruses. In contrast, the 6 internal genes of the other human isolate, A/Guangdong/SZ872/2015 (H5N6), were derived from enzootic H9N2 viruses. Although human infection has been sporadic, the co-circulation and reassortment of this virus with other enzootic low pathogenicity influenza viruses has resulted in new reassortant viruses. Further surveillance of birds is needed to monitor the spread of this novel virus.

Technical Appendix Virus isolation, sequencing methods, data sources, and description of complete genomes of 94 avian influenza viruses isolated from poultry and the environment, China.
