# Supplementary material for: Novel Reassortant Avian Influenza A(H5N6) Viruses in Humans, Guangdong, China, 2015
Source: Emerg Infect Dis. 2016 Aug;22(8):1507–9. doi: 10.3201/eid2208.160146 (PMC4982152; doi:10.3201/eid2208.160146)
Supplement: Technical Appendix — Virus isolation, sequencing methods, data sources, and description of complete genomes of 94 avian influenza viruses isolated from poultry and the environment, China. [file 16-0146-Techapp-s1.pdf]

Article DOI: http://dx.doi.org/10.3201/eid2207.160146

Novel Reassortant Avian Influenza A(H5N6) Viruses in Humans, Guangdong, China, 2015

Technical Appendix

Virus Isolation and Sequencing

On December 28, 2015, avian influenza A(H5N6) was detected in a throat swab sample from a patient (female, 26 years of age) with severe pneumonia by Shenzhen Municipal Center for Disease Control and Prevention (CDC). On December 31, 2015, another H5N6–positive case was detected in throat swab and tracheal aspirate samples from a patient (female, 40 years of age) with fever by Zhaoqing Municipal CDC. Both viruses were further confirmed to be H5N6 by full genome sequencing conducted at Guangdong Provincial CDC. Both H5N6–infected patients died; their deaths were reported to National Health and Family Planning Commission (NHFPC) of the People's Republic of China and World Health Organization (WHO) (http://www.who.int/csr/don/11-january-2016-avian-influenza-china/en/) (http://www.who.int/csr/don/4-january-2016-avian-influenza-china/en/).

Long–term avian influenza virus (AIV) surveillance was conducted in Guangdong province. We collected pairs of cloacal and oropharyngeal swab specimens from healthy poultry in several live poultry markets. Samples were stored in viral medium at 4°C until they were transported to the laboratory and then stored at –80°C until virus isolation. A 0.22–µm filter was used to sterilize the samples, which were then used to inoculate 9-day-old SPF embryonated eggs. After incubation at 37 °C for 72 h, allantoic fluid was harvested and tested by using the hemagglutination assay. Subtypes of the viruses were determined by conventional hemagglutination

inhibition and neuraminidase inhibition assays. Nine samples that tested positive for H5 and N6 were chosen for RNA extraction and sequencing in this study.

Complete genomes of the H5N6 and H6N6 AIVs were amplified by using a Qiagen OneStep RT-PCR Kit (Qiagen, Germany). PCR products were purified and sequenced on an ABI 3730XL automatic DNA analyzer. All 8 gene segments were sequenced; the nucleotide sequences were deposited into the Global Initiative on Sharing Avian Influenza Data (GISAID) (accession numbers: EPI_ISL_205959-EPI_ISL_206569).

Data Source and Analyses

All previously published H5, H6N6, and H9N2 AIV subtype sequences were collated from GenBank and the Global Initiative on Sharing Avian Influenza Data (GISAID) on 3 January 2016. Sequences of each gene segment were aligned by using MUSCLE (*1*). Phylogenetic trees were constructed by using RAxML (*2*), and the 94 sequences (Technical Appendix Table) most closely related to the H5N6 outbreak isolates were selected for further refined analyses. Maximum likelihood phylogenies were reconstructed by using PhyML with bootstrap analysis (1,000 replicates) (*3*). Evolution of virus strains is shown in online Technical Appendix Figure 2. Phylogenetic trees constructed for the HA, NA, polymerase basic-2, polymerase basic-1 (PB1), polymerase acidic (PA), nucleoprotein (NP), matrix (M), and nonstructural (NS) segments are shown in online Technical Appendix Figure 1.

References

1. Edgar RC. MUSCLE: multiple sequence alignment with high accuracy and high throughput. Nucleic Acids Res. 2004;32:1792–7. http://dx.doi.org/10.1093/nar/gkh340

2. Stamatakis A. RAxML-VI-HPC: maximum likelihood-based phylogenetic analyses with thousands of taxa and mixed models. Bioinformatics. 2006;22:2688–90. http://dx.doi.org/10.1093/bioinformatics/btl446

3. Guindon S, Dufayard JF, Lefort V, Anisimova M, Hordijk W, Gascuel O. New algorithms and methods to estimate maximum-likelihood phylogenies: assessing the performance of PhyML 3.0. Syst Biol. 2010;59:307–21. http://dx.doi.org/10.1093/sysbio/syq010

Technical Appendix Table. Complete genomes of 94 avian influenza viruses isolated from poultry and the environment, China*

Strain name	Genbank accession numbers							
	PB2	PB1	PA	HA	NP	NA	M	NSP
A/Anas crecca/Hubei/Chenhu1623–5/2014 (H5N6)	KM251532	KM251522	KM251512	KM251462	KM251492	KM251485	KM251472	KM251502
A/black chicken/Jiangxi/10129/2014 (H5N6)	KP286450	KP286451	KP286452	KP286453	KP286454	KP286455	KP286456	KP286457
A/cat/Sichuan/SC18/2014 (H5N6)	KM873635	KM873636	KM873637	KM873638	KM873639	KM873640	KM873641	KM873642
A/chicken/DaLi/302/2014 (H5N1)	KP732559	KP732560	KP732561	KM392379	KP732562	KP732563	KP732564	KP732565
A/chicken/Dongguan/2690/2013 (H5N6)	KP286098	KP286099	KP286100	KP286101	KP286102	KP286103	KP286104	KP286105
A/chicken/Dongguan/3056/2013 (H9N2)	KP414562	KP414563	KP414564	KP414565	KP414566	KP414567	KP414568	KP414569
A/chicken/Dongguan/3363/2013 (H5N6)	KP286106	KP286107	KP286108	KP286109	KP286110	KP286111	KP286112	KP286113
A/chicken/Dongguan/3964/2013 (H9N2)	KP414642	KP414643	KP414644	KP414645	KP414646	KP414647	KP414648	KP414649
A/chicken/Dongguan/4189/2013 (H9N2)	KP414666	KP414667	KP414668	KP414669	KP414670	KP414671	KP414672	KP414673
A/chicken/Dongguan/4259/2013 (H5N6)	KP285026	KP285027	KP285028	KP285029	KP285030	KP285031	KP285032	KP285033
A/chicken/Hong Kong/NT266/2012 (H9N2)	KF260899	KF260655	KF260411	KF259157	KF259923	KF259559	KF259404	KF260167
A/chicken/Hubei/2014 (H9N2)	KT164848	KT164849	KT164850	KT164851	KT164852	KT164853	KT164854	KT164855
A/chicken/Jiangxi/13240/2014 (H10N6)	KP286346	KP286347	KP286348	KP286349	KP286350	KP286351	KP286352	KP286353
A/chicken/Jiangxi/29117/2013 (H9N2)	KP285298	KP285299	KP285300	KP285301	KP285302	KP285303	KP285304	KP285305
A/chicken/Jiangxi/34682/2013 (H9N2)	KP285458	KP285459	KP285460	KP285461	KP285462	KP285463	KP285464	KP285465
A/chicken/Jiangxi/NCDZT1123/2014 (H5N6)	KP090436	KP090437	KP090438	KP090439	KP090440	KP090441	KP090442	KP090443
A/chicken/Jiangxi/NCDZT1126/2014 (H5N6)	KP090444	KP090445	KP090446	KP090447	KP090448	KP090449	KP090450	KP090451
A/chicken/Laos/LPQ001/2014 (H5N6)	KM496962	KM496963	KM496964	KM496965	KM496966	KM496967	KM496968	KM496969
A/chicken/Qingdao/013/2014 (H9N2)	KT449724	KT449704	KT449684	KT449584	KT449644	KT449624	KT449604	KT449664
A/chicken/Rizhao/55/2013 (H9N2)	KF260941	KF260697	KF260453	KF259166	KF259965	KF259569	KF259446	KF260209
A/chicken/Shanghai/SC387/2013 (H9N2)	KM113050	KM113051	KM113052	KM113057	KM113054	KM113055	KM113056	KM113053
A/chicken/Shenzhen/1061/2013 (H5N6)	KP286082	KP286083	KP286084	KP286085	KP286086	KP286087	KP286088	KP286089
A/chicken/Shenzhen/1395/2013 (H5N6)	KP284970	KP284971	KP284972	KP284973	KP284974	KP284975	KP284976	KP284977
A/chicken/Shenzhen/1488/2013 (H9N2)	KP414378	KP414379	KP414380	KP414381	KP414382	KP414383	KP414384	KP414385
A/chicken/Shenzhen/1799/2013 (H9N2)	KP414426	KP414427	KP414428	KP414429	KP414430	KP414431	KP414432	KP414433
A/chicken/Shenzhen/1845/2013 (H5N6)	KP284978	KP284979	KP284980	KP284981	KP284982	KP284983	KP284984	KP284985
A/chicken/Shenzhen/2269/2013 (H5N6)	KP286090	KP286091	KP286092	KP286093	KP286094	KP286095	KP286096	KP286097
A/chicken/Shenzhen/552/2013 (H5N6)	KP284962	KP284963	KP284964	KP284965	KP284966	KP284967	KP284968	KP284969
A/chicken/Shenzhen/715/2013 (H5N6)	KP286074	KP286075	KP286076	KP286077	KP286078	KP286079	KP286080	KP286081
A/chicken/Sichuan/NCJPL1/2014 (H5N6)	KM251533	KM251523	KM251513	KM251463	KM251493	KM251486	KM251473	KM251503
A/chicken/Taizhou/TZJF05/2015 (H9N2)	KU143584	KU143541	KU143498	KU143287	KU143412	KU143351	KU143326	KU143455
A/chicken/Wenzhou/YHQL04/2014 (H9N2)	KU143582	KU143539	KU143496	KU143285	KU143410	KU143349	KU143324	KU143453
A/chicken/Wuhan/WHYJ02/2015 (H5N6)	KU143578	KU143535	KU143492	KU143268	KU143403	KU143364	KU143319	KU143449
A/chicken/Zhejiang/6C2/2013 (H5N6)	KJ807774	KJ807775	KJ807777	KJ807779	KJ807781	KJ807783	KJ807785	KJ807787
A/chicken/Zhejiang/727079/2014_H5N2	KU042667	KU042695	KU042724	KU042753	KU042782	KU042811	KU042840	KU042869
A/chicken/Zhejiang/727155/2014 (H5N6)	KU042679	KU042708	KU042737	KU042766	KU042795	KU042824	KU042853	KU042882
A/chicken/Zhejiang/727192/2014 (H9N2)	KU042521	KU042539	KU042557	KU042575	KU042593	KU042611	KU042629	KU042647
A/chicken/Zhejiang/727198/2014 (H9N2)	KU042522	KU042540	KU042558	KU042576	KU042594	KU042612	KU042630	KU042648
A/chicken/Zhejiang/925117/2014 (H9N2)	KU042525	KU042543	KU04256	KU042579	KU042597	KU042615	KU042633	KU042651
A/chicken/Zhejiang/925134/2014 (H9N2)	KU042527	KU042545	KU042563	KU042581	KU042599	KU042617	KU042635	KU042653
A/duck/Dongguan/3069/2013 (H5N6)	KP285018	KP285019	KP285020	KP285021	KP285022	KP285023	KP285024	KP285025
A/duck/Fujian/5474/2007 (H6N6)	CY109863	CY109864	CY109865	CY109866	CY109867	CY109868	CY109869	CY109870
A/duck/Guangdong/GD01/2014 (H5N6)	KJ754142	KJ754143	KJ754144	KJ754145	KJ754146	KJ754147	KJ754148	KJ754149
A/duck/Hunan/S4273/2010 (H6N6)	KJ200892	KJ200891	KJ200890	KJ200885	KJ200888	KJ200887	KJ200886	KJ200889
A/duck/Jiangsu/k1203/2010 (H5N8)	JQ973691	JQ973692	JQ973693	JQ973694	JQ973695	JQ973696	JQ973697	JQ973698
A/duck/Jiangxi/10160/2014 (H5N6)	KP285034	KP285035	KP285036	KP285037	KP285038	KP285039	KP285040	KP285041

Strain name	Genbank accession numbers							
	PB2	PB1	PA	HA	NP	NA	M	NSP
A/duck/Jiangxi/13469/2014 (H5N6)	KP286418	KP286419	KP286420	KP286421	KP286422	KP286423	KP286424	KP286425
A/duck/Laos/XBY004/2014 (H5N6)	KM496974	KM496975	KM496976	KM496977	KM496978	KM496979	KM496980	KM496981
A/duck/Sichuan/NCXJ15/2014 (H5N6)	KM251535	KM251525	KM251515	KM251465	KM251495	KM251488	KM251475	KM251505
A/duck/Sichuan/NCXN11/2014 (H5N1)	KM251540	KM251530	KM251520	KM251470	KM251500	KM251483	KM251480	KM251510
A/duck/Vietnam/LBM360c1–4-1/2013 (H5N6)	LC010693	LC010694	LC010695	LC010696	LC010697	LC010698	LC010699	LC010700
A/duck/Vietnam/LBM473/2013 (H6N6)	AB916656	AB916657	AB916658	AB916659	AB916660	AB916661	AB916662	AB916663
A/duck/Vietnam/LBM639/2014 (H5N1)	AB979508	AB979509	AB979510	AB979511	AB979512	AB979513	AB979514	AB979515
A/duck/Wenzhou/YHQL64/2014 (H9N2)	KU143583	KU143540	KU143497	KU143286	KU143411	KU143350	KU143325	KU143454
A/duck/Wuhan/JXYFB22/2015 (H5N6)	KU143579	KU143536	KU143493	KU143270	KU143409	KU143368	KU143321	KU143451
A/duck/Wuhan/WHYF02/2015 (H5N6)	KU143575	KU143532	KU143489	KU143265	KU143402	KU143365	KU143318	KU143446
A/duck/Yamagata/061004/2014 (H6N6)	LC042078	LC042079	LC042080	LC042081	LC042082	LC042083	LC042084	LC042085
A/duck/Zhejiang/6D2/2013 (H5N6)	KJ807773	KJ807776	KJ807778	KJ807780	KJ807782	KJ807784	KJ807786	KJ807788
A/duck/Zhejiang/727041/2014 (H5N2)	KU042662	KU042691	KU042720	KU042749	KU042778	KU042807	KU042836	KU042865
A/duck/Zhejiang/727158/2014 (H5N6)	KU042668	KU042697	KU042726	KU042755	KU042784	KU042813	KU042842	KU042871
A/environment/Guangdong/HY243/2015 (H5N6)	KT370112	KT370104	KT370097	KT370063	KT370085	KT370072	KT370070	KT370090
A/environment/Guangdong/HZ058/2013 (H6N6)	KT370052	KT370043	KT370033	KT369984	KT370013	KT370002	KT369994	KT370022
A/environment/Guangdong/ZS356/2014 (H5N6)	KT370107	KT370100	KT370093	KT370061	KT370080	KT370078	KT370065	KT370086
A/environment/Yunnan/YN19/2015 (H5N6)	ND	KT963052	KT963051	KT963046	KT963049	KT963048	KT963047	KT963050
A/environment/Yunnan/YN22/2015 (H5N6)	KT963060	KT963059	KT963058	KT963053	KT963056	KT963055	KT963054	KT963057
A/goose/Shantou/1763/2014 (H5N6)	KP285314	KP285315	KP285316	KP285317	KP285318	KP285319	KP285320	KP285321
A/goose/Zhejiang/112080/2014 (H5N6)	KU042675	KU042704	KU042733	KU042762	KU042791	KU042820	KU042849	KU042878
A/goose/Zhejiang/77168/2014_H5N2	KU042665	KU042694	KU042723	KU042752	KU042781	KU042810	KU042839	KU042868
A/goose/Zhejiang/925036/2014 (H5N6)	KU042671	KU042700	KU042729	KU042758	KU042787	KU042816	KU042845	KU042874
A/goose/Zhejiang/925108/2014 (H5N6)	KU042674	KU042703	KU042732	KU042761	KU042790	KU042819	KU042848	KU042877
A/Guangzhou/39715/2014 (H5N6)	KP765785	KP765786	KP765787	KP765788	KP765789	KP765790	KP765791	KP765792
A/silkie chicken/Dongguan/2809/2013 (H5N6)	KP285010	KP285011	KP285012	KP285013	KP285014	KP285015	KP285016	KP285017
A/swan goose/Jilin/JL01/2014 (H5N6)	KM873643	KM873644	KM873645	KM873646	KM873647	KM873648	KM873649	KM873650
A/swine/Guangdong/1/2014 (H5N6)	KT313409	KT313408	KT313407	KT313418	KT313405	KT313404	KT313403	KT313406
A/swine/Guangdong/2/2014 (H5N6)	KT313417	KT313416	KT313415	KT313410	KT313413	KT313412	KT313411	KT313414
A/turtledove/Wuhan/HKBJ07/2015 (H5N6)	KU143569	KU143527	KU143484	KU143273	KU143408	KU143358	KU143312	KU143440
A/turtledove/Wuhan/HKBJ62/2015 (H6N6)	KU143558	KU143515	KU143472	KU143274	KU143386	KU143356	KU143300	KU143429
A/turtledove/Wuhan/WHBJ12/2014 (H5N6)	KU143570	KU143528	KU143485	KU143261	KU143397	KU143359	KU143313	KU143441
A/wild chicken/Shanghai/C1/2014 (H9N2)	KJ726736	KJ726730	KJ726724	KJ726694	KJ726712	KJ726706	KJ726700	KJ726718
A/wild duck/Shandong/628/2011 (H5N1)	JX534562	JX534563	JX534564	JX534565	JX534566	JX534567	JX534568	JX534569
A/Yunnan/0127/2015 (H5N6)	KT245150	KT245149	KT245148	KT245143	KT245146	KT245145	KT245144	KT245147
A/Hong Kong/308/2014 (H9N2)	EPI498034	EPI498035	EPI498033	EPI498037	EPI498030	EPI498036	EPI498032	EPI498031
A/Sichuan/26221/2014 (H5N6)	EPI533585	EPI533586	EPI533587	EPI533583	EPI533588	EPI533584	EPI533589	EPI533590
A/environment/Guangdong/SS127/2013 (H5N6)†	EPI_ISL_205965							
A/goose/Guangdong /JG125/2013 (H5N6)†	EPI_ISL_205961							
A/goose/Guangdong /SSLBY/2015 (H5N6)†	EPI_ISL_205966							
A/goose/Guangdong /SSZYP/2015 (H5N6)†	EPI_ISL_205967							
A/chicken/Guangdong/FG594/2015 (H5N6)†	EPI_ISL_205964							
A/duck/Guangdong/PY501/2014 (H5N6)†	EPI_ISL_205962							
A/duck/Guangdong/PY503/2014 (H5N6)†	EPI_ISL_205963							
A/duck/Guangdong/SS1A1/2014 (H5N6)†	EPI_ISL_205959							
A/Guangdong/SZ872/2015 (H5N6)†	EPI_ISL_206568							
A/Guangdong/ZQ874/2015 (H5N6)†	EPI_ISL_206569							
A/Chicken/Guangdong/PY480/2014 (H6N6)†	EPI_ISL_205960							

Strain name	Genbank accession numbers							
	PB2	PB1	PA	HA	NP	NA	M	NSP

*PB, polymerase basic; PA, polymerase; HA, hemagglutinin; NP, nucleoprotein; NA, neuraminidase; M, matrix; NSP, nonstructural protein; ND, no data.

†The complete genomes of these viruses were deposited in the Global Initiative on Sharing Avian Influenza Data (GISAID) EpiFlu database (http://platform.gisaid.org).

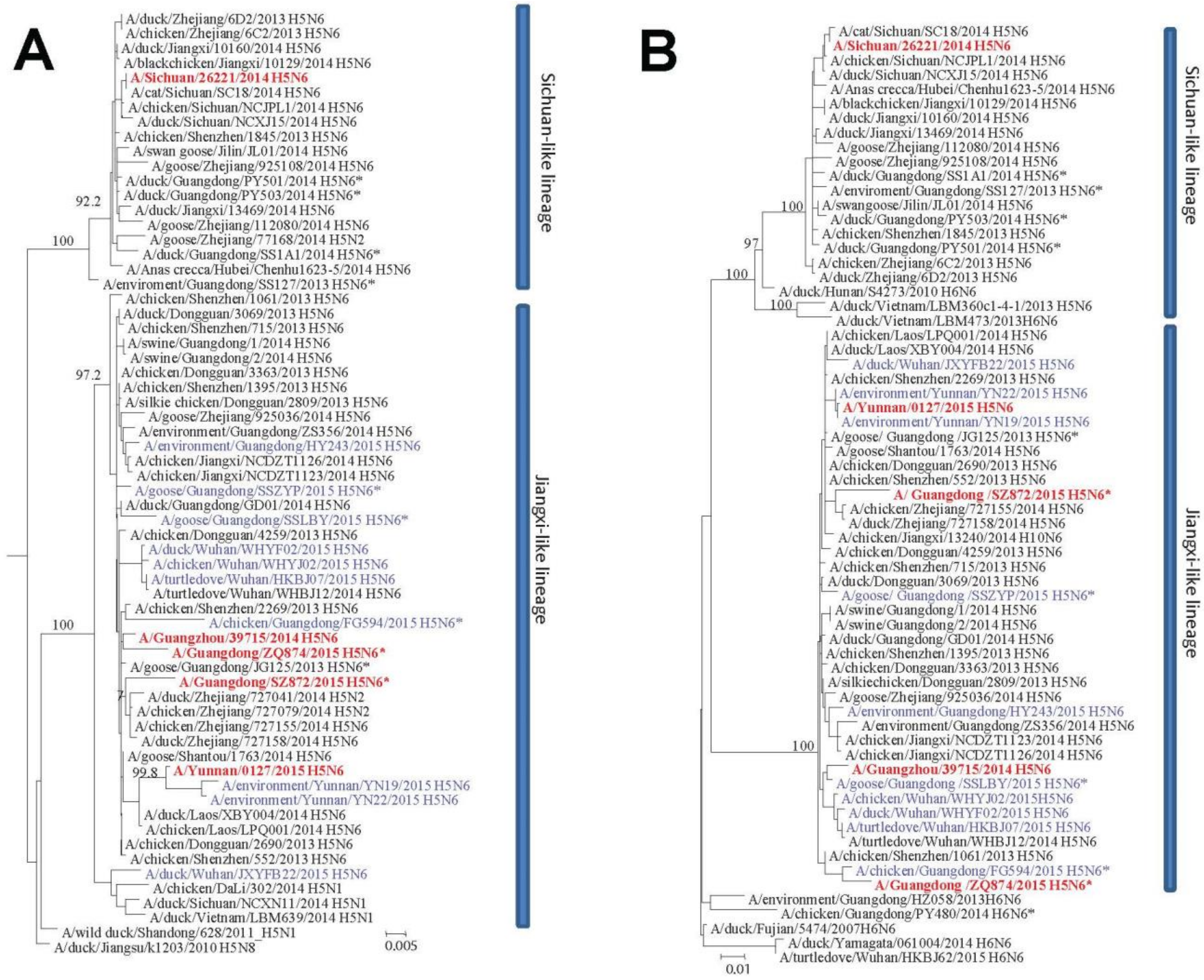

A
B
Sichuan-like lineage
Jiangxi-like lineage
A/Sichuan/26221/2014 H5N6
A/Guangzhou/39715/2014 H5N6
A/Guangdong/ZQ874/2015 H5N6*
A/Guangdong/SZ872/2015 H5N6*
A/Yunnan/0127/2015 H5N6
0.005
0.01

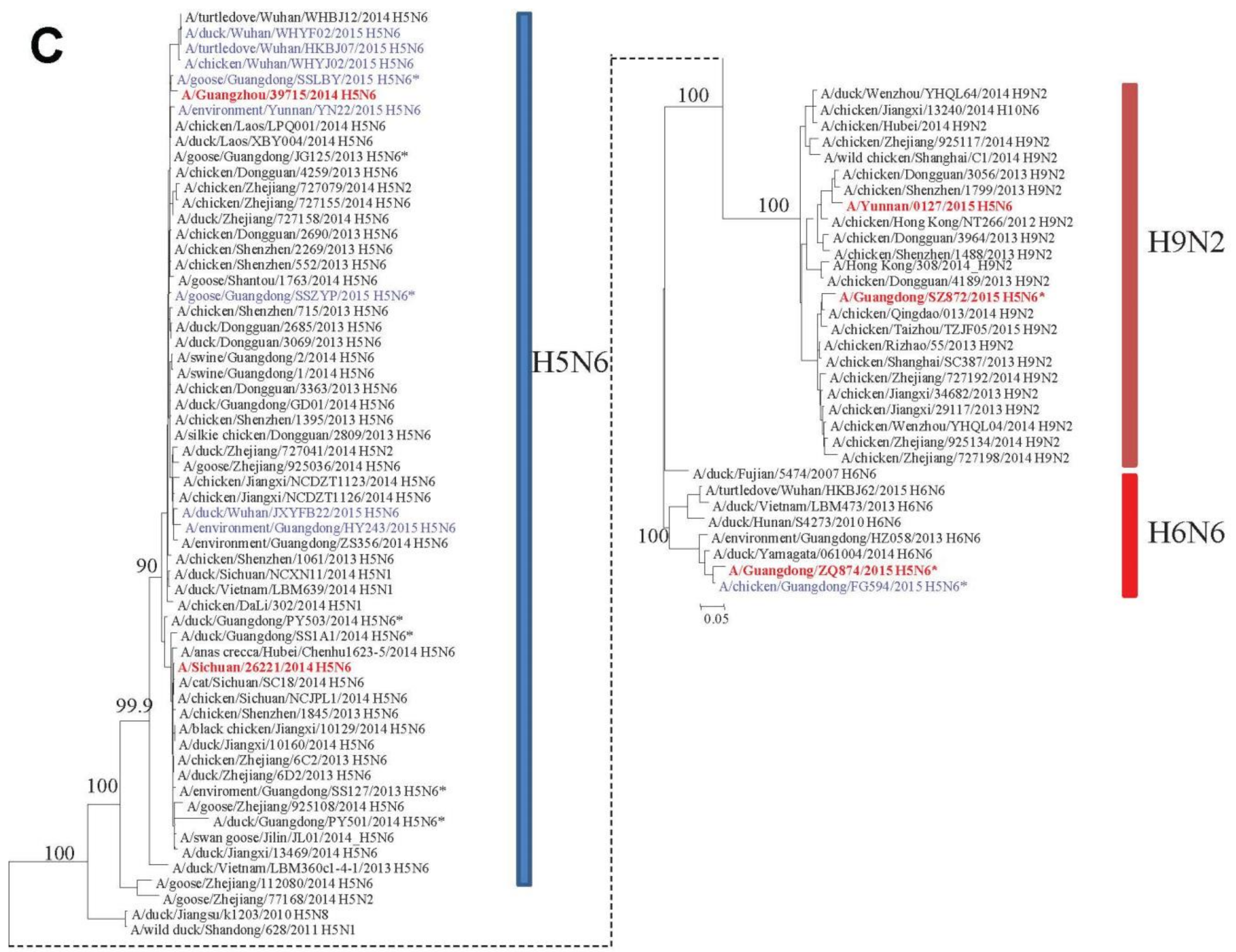
C
A/turtledove/Wuhan/WHBJ12/2014 H5N6
A/duck/Wuhan/WHYF02/2015 H5N6
A/turtledove/Wuhan/HKBJ07/2015 H5N6
A/chicken/Wuhan/WHYJ02/2015 H5N6
A/goose/Guangdong/SSLBY/2015 H5N6*
A/Guangzhou/39715/2014 H5N6
A/environment/Yunnan/YN22/2015 H5N6
A/chicken/Laos/LPQ001/2014 H5N6
A/duck/Laos/XBY004/2014 H5N6
A/goose/Guangdong/JG125/2013 H5N6*
A/chicken/Dongguan/4259/2013 H5N6
A/chicken/Zhejiang/727079/2014 H5N2
A/chicken/Zhejiang/727155/2014 H5N6
A/duck/Zhejiang/727158/2014 H5N6
A/chicken/Dongguan/2690/2013 H5N6
A/chicken/Shenzhen/2269/2013 H5N6
A/chicken/Shenzhen/552/2013 H5N6
A/goose/Shantou/1763/2014 H5N6
A/goose/Guangdong/SSZYP/2015 H5N6*
A/chicken/Shenzhen/715/2013 H5N6
A/duck/Dongguan/2685/2013 H5N6
A/duck/Dongguan/3069/2013 H5N6
A/swine/Guangdong/2/2014 H5N6
A/swine/Guangdong/1/2014 H5N6
A/chicken/Dongguan/3363/2013 H5N6
A/duck/Guangdong/GD01/2014 H5N6
A/chicken/Shenzhen/1395/2013 H5N6
A/silkie chicken/Dongguan/2809/2013 H5N6
A/duck/Zhejiang/727041/2014 H5N2
A/goose/Zhejiang/925036/2014 H5N6
A/chicken/Jiangxi/NCDZT1123/2014 H5N6
A/chicken/Jiangxi/NCDZT1126/2014 H5N6
A/duck/Wuhan/JXYFB22/2015 H5N6
A/environment/Guangdong/HY243/2015 H5N6
A/environment/Guangdong/ZS356/2014 H5N6
90
A/chicken/Shenzhen/1061/2013 H5N6
A/duck/Sichuan/NCXN11/2014 H5N1
A/duck/Vietnam/LBM639/2014 H5N1
A/chicken/DaLi/302/2014 H5N1
A/duck/Guangdong/PY503/2014 H5N6*
A/duck/Guangdong/SS1A1/2014 H5N6*
A/anas crecca/Hubei/Chenhu1623-5/2014 H5N6
A/Sichuan/26221/2014 H5N6
A/cat/Sichuan/SC18/2014 H5N6
99.9
A/chicken/Sichuan/NCJPL1/2014 H5N6
A/chicken/Shenzhen/1845/2013 H5N6
A/black chicken/Jiangxi/10129/2014 H5N6
A/duck/Jiangxi/10160/2014 H5N6
A/chicken/Zhejiang/6C2/2013 H5N6
100
A/duck/Zhejiang/6D2/2013 H5N6
A/enviroment/Guangdong/SS127/2013 H5N6*
A/goose/Zhejiang/925108/2014 H5N6
A/duck/Guangdong/PY501/2014 H5N6*
A/swan goose/Jilin/JL01/2014_H5N6
100
A/duck/Jiangxi/13469/2014 H5N6
A/duck/Vietnam/LBM360c1-4-1/2013 H5N6
A/goose/Zhejiang/112080/2014 H5N6
A/goose/Zhejiang/77168/2014 H5N2
A/duck/Jiangsu/k1203/2010 H5N8
A/wild duck/Shandong/628/2011 H5N1
H5N6
100
A/duck/Wenzhou/YHQL64/2014 H9N2
A/chicken/Jiangxi/13240/2014 H10N6
A/chicken/Hubei/2014 H9N2
A/chicken/Zhejiang/925117/2014 H9N2
A/wild chicken/Shanghai/C1/2014 H9N2
A/chicken/Dongguan/3056/2013 H9N2
A/chicken/Shenzhen/1799/2013 H9N2
100
A/Yunnan/0127/2015 H5N6
A/chicken/Hong Kong/NT266/2012 H9N2
A/chicken/Dongguan/3964/2013 H9N2
A/chicken/Shenzhen/1488/2013 H9N2
A/Hong Kong/308/2014_H9N2
A/chicken/Dongguan/4189/2013 H9N2
A/Guangdong/SZ872/2015 H5N6*
A/chicken/Qingdao/013/2014 H9N2
A/chicken/Taizhou/TZJF05/2015 H9N2
A/chicken/Rizhao/55/2013 H9N2
A/chicken/Shanghai/SC387/2013 H9N2
A/chicken/Zhejiang/727192/2014 H9N2
A/chicken/Jiangxi/34682/2013 H9N2
A/chicken/Jiangxi/29117/2013 H9N2
A/chicken/Wenzhou/YHQL04/2014 H9N2
A/chicken/Zhejiang/925134/2014 H9N2
A/chicken/Zhejiang/727198/2014 H9N2
H9N2
A/duck/Fujian/5474/2007 H6N6
A/turtledove/Wuhan/HKBJ62/2015 H6N6
A/duck/Vietnam/LBM473/2013 H6N6
A/duck/Hunan/S4273/2010 H6N6
100
A/environment/Guangdong/HZ058/2013 H6N6
A/duck/Yamagata/061004/2014 H6N6
A/Guangdong/ZQ874/2015 H5N6*
A/chicken/Guangdong/FG594/2015 H5N6*
H6N6
0.05

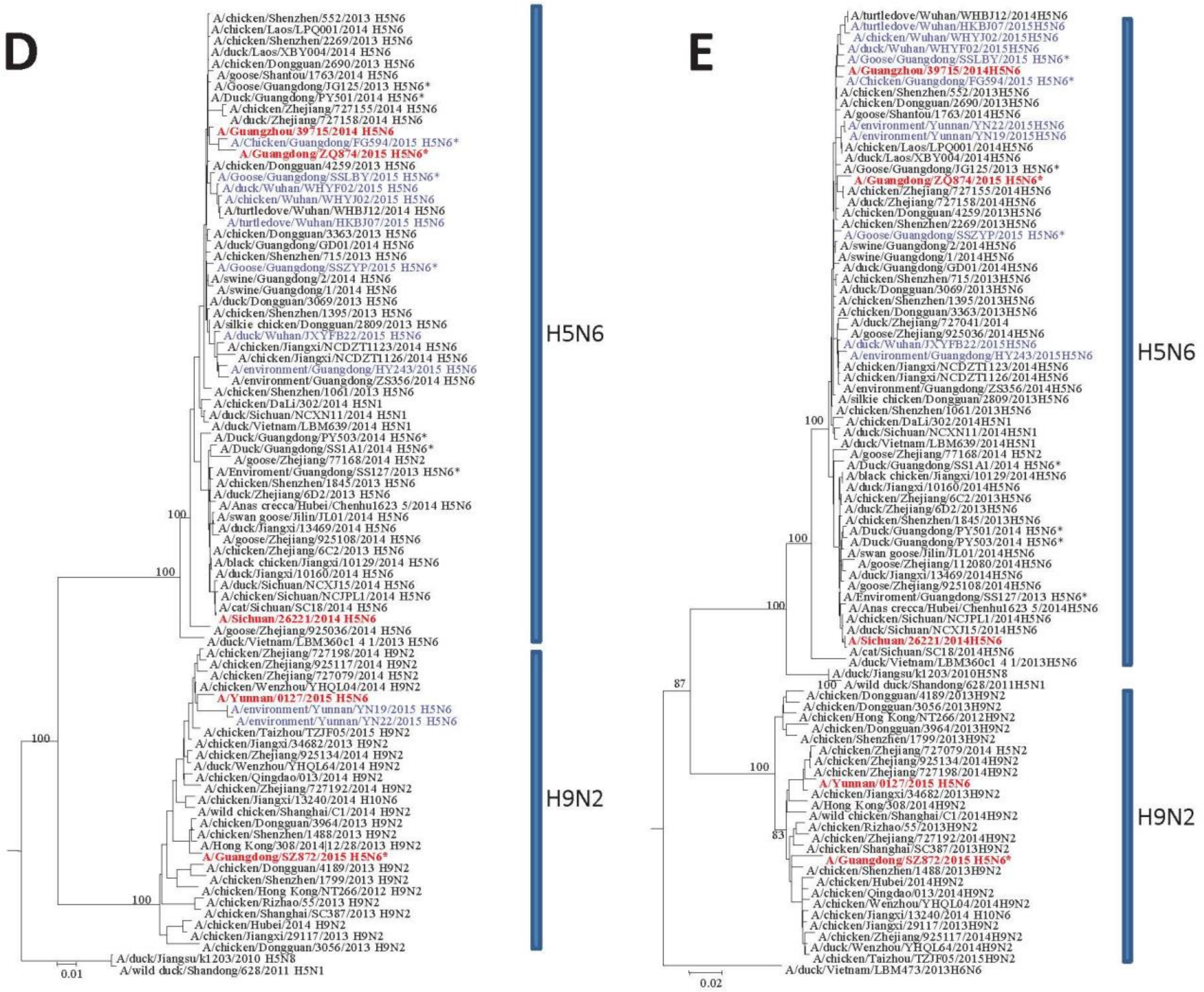
D
E
H5N6
H9N2
H5N6
H9N2
100
100
100
100
0.01
87
100
100
100
100
100
83
0.02

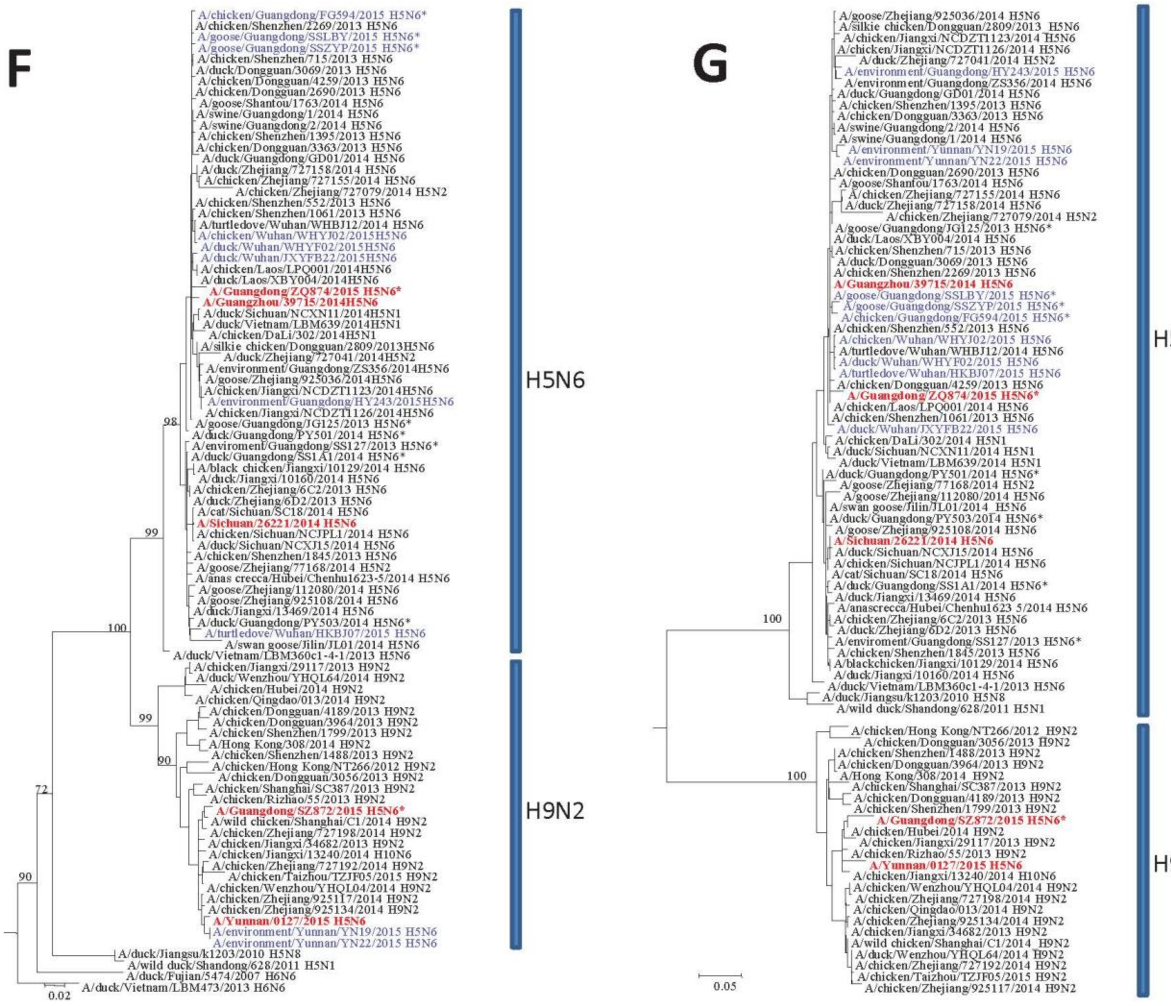
F
H5N6
H9N2
0.02
G
H5N6
H9N2
0.05

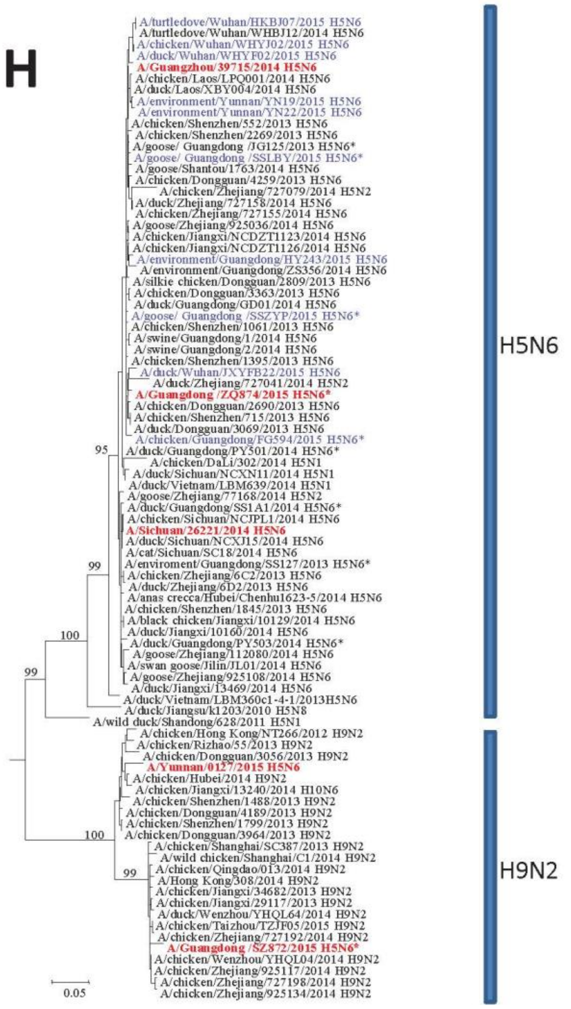

Technical Appendix Figure 1. Phylogenetic relationships of H5N6 influenza viruses. A) hemagglutinin (HA); B) neuraminidase (NA); C) polymerase basic-2 (PB2); D) polymerase basic-1 (PB1); E) polymerase acidic (PA); F) nucleoprotein (NP); G) matrix (M); H) non-structural (NS). Maximum-likelihood trees were constructed using PhyML. Bootstrap values were calculated on 1,000 replicates. Viruses that infect humans are indicated in red. H5N6 viruses isolated in 2015 are indicated in blue; viruses sequenced in this study were marked with *.

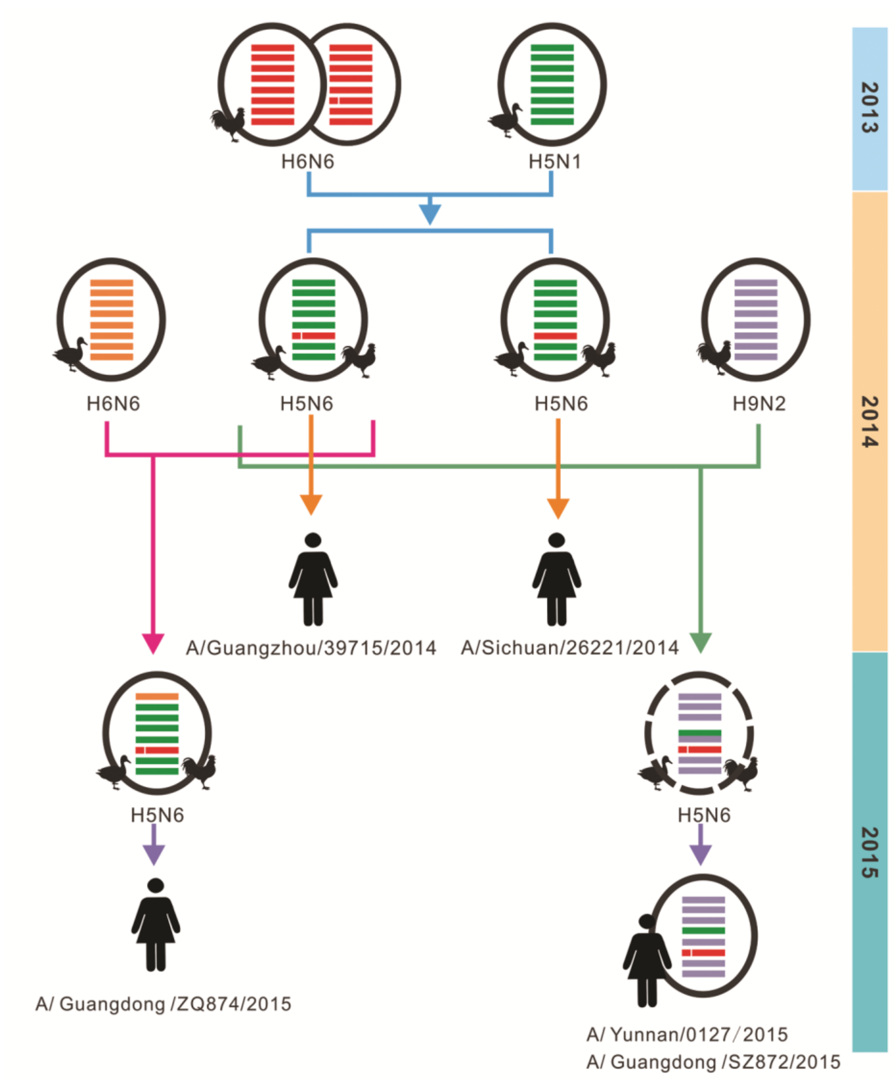

Technical Appendix Figure 2. Hypothetical lineage for the origin of the novel reassortant avian influenza A(H5N6) viruses in humans, Guangdong, China, 2015. Virus particles are represented by ovals containing horizontal bars that represent the 8 gene segments (from top to bottom: polymerase basic 2, polymerase basic 1, polymerase acidic protein, hemagglutinin, nucleocapsid protein, neuraminidase, matrix protein, and nonstructural protein). A broken bar representing neuraminidase (segment 6) indicates an 11 aa deletion in the stalk region. Virus indicated by a broken oval represents a hypothetical reassortant. Source viruses for a reassortant virus are adjacent to the arrow tails; arrow heads point to the resulting reassortant viruses. Colored bars indicate poultry source and year of original isolation: red: chicken, 2013; green: duck, 2013; orange: duck, 2014; purple: chicken, 2014.
